# Surfactant Partitioning Dynamics in Freshly Generated Aerosol Droplets

**DOI:** 10.1021/jacs.4c03041

**Published:** 2024-06-01

**Authors:** Alison Bain, Lara Lalemi, Nathan Croll Dawes, Rachael E. H. Miles, Alexander M. Prophet, Kevin R. Wilson, Bryan R. Bzdek

**Affiliations:** † School of Chemistry, University of Bristol, Cantock’s Close, Bristol BS8 1TS, U.K.; ‡ Department of Chemistry, Oregon State University, Corvallis, Oregon 97331, United States; § Department of Chemistry, University of California, Berkeley, California 94720, United States; ∥ Chemical Sciences Division, Lawrence Berkeley National Laboratory, Berkeley, California 94720, United States

## Abstract

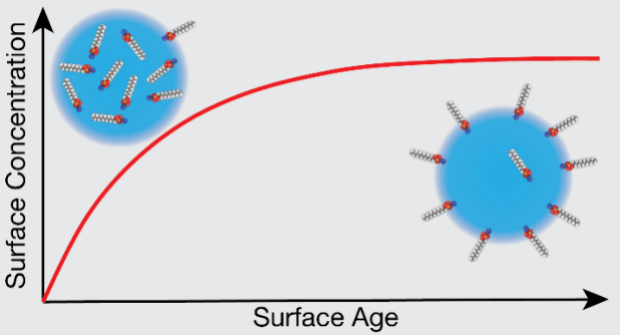

Aerosol droplets are unique microcompartments with relevance to areas as diverse as materials and chemical synthesis, atmospheric chemistry, and cloud formation. Observations of highly accelerated and unusual chemistry taking place in such droplets have challenged our understanding of chemical kinetics in these microscopic systems. Due to their large surface-area-to-volume ratios, interfacial processes can play a dominant role in governing chemical reactivity and other processes in droplets. Quantitative knowledge about droplet surface properties is required to explain reaction mechanisms and product yields. However, our understanding of the compositions and properties of these dynamic, microscopic interfaces is poor compared to our understanding of bulk processes. Here, we measure the dynamic surface tensions of 14–25 μm radius (11–65 pL) droplets containing a strong surfactant (either sodium dodecyl sulfate or octyl-β-D-thioglucopyranoside) using a stroboscopic imaging approach, enabling observation of the dynamics of surfactant partitioning to the droplet–air interface on time scales of 10s to 100s of microseconds after droplet generation. The experimental results are interpreted with a state-of-the-art kinetic model accounting for the unique high surface-area-to-volume ratio inherent to aerosol droplets, providing insights into both the surfactant diffusion and adsorption kinetics as well as the time-dependence of the interfacial surfactant concentration. This study demonstrates that microscopic droplet interfaces can take up to many milliseconds to reach equilibrium. Such time scales should be considered when attempting to explain observations of accelerated chemistry in microcompartments.

## Introduction

The interfaces of microscopic aerosol droplets greatly impact their chemical and physical properties.^1−3^ For instance, the surface tension of atmospheric aerosol droplets (which is directly related to their surface composition) is weakly constrained but strongly influences the probability of activation to form cloud droplets and impact climate.^4−10^ Surface tension can also control the morphology of spray-dried particles^11,12^ widely used in many industrial applications. Finally, unique chemistry at the droplet–air interface is often invoked to explain observations of significantly accelerated (factors of 10^2^–10^6^) or unexpected chemical reactions in aerosol droplets and other microcompartments compared to within macroscopic solutions.^13−25^

The role of the interface in driving this accelerated chemistry is often poorly understood,^26−28^ but resolving interfacial properties becomes increasingly important in aerosols and droplets.^29^ These microscopic compartments contain significantly more surface area relative to their volume compared to a macroscopic solution.^30^ For example, a liter of solution atomized to form 1 μm-diameter droplets contains approximately 10^5^ times more total surface area, increasing the importance of interfacial relative to bulk chemistry. Moreover, an aerosol droplet’s large interfacial area can perturb the bulk concentration of molecules with some surface propensity, as a significant fraction of these molecules will be partitioned to the interface, thereby depleting the droplet’s bulk concentration.^24,31−34^ Consequently, chemical reactions become more sensitive to partitioning equilibria at the interface.^23,35^ Reaction rates that may be accelerated at the droplet–air interface will depend on the interfacial concentration of the reactants, thus requiring knowledge about the interplay between the adsorption and desorption rates at the interface and the diffusion rate in the droplet bulk. Indeed, surfactants can in some cases accelerate^36^ and, in other cases, inhibit^18^ compartmentalized reactivity.

Since reactions can undergo unique interfacial pathways in droplets, developing accurate mechanisms to predict chemical reactivity requires measurements of aerosol droplet surface properties. Although some approaches are capable of resolving near-equilibrium surface tensions^37−41^ or surface compositions^42−45^ of aerosol droplets, observing the dynamics is more challenging, particularly because the dynamics can occur on microsecond time scales. Macroscopic solution measurements of dynamic surface tension often utilize bubble pressure or pendant drop tensiometry.^7,46,47^ However, the interfaces typically investigated with these experiments correspond to millimeter-sized droplets, and measurements are limited to time scales longer than ∼10 ms (much slower than the time scales of chemical reactions accelerated in microdroplets).^14,18,48,49^ Because these approaches are not sensitive to the unique aspects of high surface-area-to-volume ratio droplets,^33,50−52^ they are not suitable for extrapolation to microscopic systems. Hence, methods capable of investigating dynamic surface properties at the microdroplet level are required.

Some efforts have been made toward this end, mainly by stroboscopically imaging oscillating droplets to retrieve their surface tension. For instance, Stückrad et al. measured the dynamic surface tension of aqueous heptanol droplets with radii >170 μm (∼20 nL).^53^ The large radius implies these droplets can be treated as macroscopic systems, and the authors modeled the observed dynamics with the Ward–Tordai description of surface tension dynamics to a planar interface^54^ using the Frumkin equation of state. Later, Staat et al. stroboscopically imaged aqueous sodium dodecyl sulfate (SDS) droplets at two radii, 1.2 mm (7 μL, measurement window of >40 ms) and 34 μm (165 pL, measurement window on the order of 100s of μs).^55^ Although the equilibrium surface tension was retrieved for the larger droplet, the 34 μm droplet’s surface tension was much higher than the equilibrium value, suggesting it had not yet reached its equilibrium surface composition during the measurement window. However, dynamic changes in the surface tension of the 34 μm droplet were not resolved. Moreover, neither study modeled the dynamics in a manner that could be extended to smaller aerosol droplets where interfacial dynamics are distinct from those in macroscopic solutions.

In this contribution, we measure the dynamic surface tension of surfactant-containing droplets with radii 14–25 μm (11–65 pL) using a stroboscopic imaging approach. A droplet’s dynamic surface tension is resolved with up to 6 μs time resolution over a measurement window spanning ∼10–500 μs after droplet generation. A kinetic model accounting for the high surface-area-to-volume ratio in droplets is applied and used to predict the time-dependence of surfactant partitioning. The results reasonably match the experimental observations, providing insight into the factors controlling surfactant partitioning in picoliter volumes. Quantitative knowledge of partitioning dynamics to microscopic interfaces has direct impacts on predicting how a droplet’s chemical and physical properties evolve during chemical reaction.

## Experimental Section

### Measuring Surface Tension Dynamics in Microscopic Droplets

#### Chemicals

Solutions of sodium dodecyl sulfate (SDS, MP, ultrapure) and octyl-β-D-thioglucopyranoside (OTG, Sigma >98.0% purity) were made with deionized water. The SDS was found to contain surface-active impurities (observed as a dip in the equilibrium surface tension after the critical micelle concentration (CMC) was reached/before the surface tension plateaued). To remove the impurities, SDS was recrystallized three times in ethanol before making solutions. After purification, the equilibrium surface tensions and CMC for aqueous SDS agreed with the literature values.^56^

#### Macroscopic Solution Surface Tension Measurements

Macroscopic equilibrium surface tension measurements (σ, in eq 1) were collected using the Wilhelmy plate method (K100, Krüss) and fit with the Langmuir isotherm (eq 1) and equation of state (eq 2) as a function of surfactant concentration, [surf_(bulk)_], where Θ is the fractional surface coverage. This fitting procedure enabled determination of the maximum surface excess (Γ_∞_) and equilibrium partitioning constant (*K*_eq_^surf^) using the surface tension of water σ_0_ = 72.8 mN/m, gas constant (*R*), temperature *T* = 298 K, and *n* = 1 for nonionic surfactants or *n* = 2 for ionic surfactants. Retrieved parameters from these fits are provided in Table 1, and the experimental data and isotherm fits for SDS and OTG^31^ are shown in Figure S1.
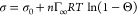
1
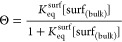
2

**Table 1:** Langmuir Isotherm Fit Parameters and Kinetic Parameters Used in the Kinetiscope Model^a^

	*n*^b^	Γ_∞_^b^ (mol/m^2^)	[Site]_max_^c^ (molecules/cm^3^)	*K*_eq_^surf^ (m^3^/mol)^b^	*k*_solv_^d^ (s^–1^)	*k*_desolv_^e^ (m^3^/mol·s)	*D*_apparent_^d^ (m^2^/s)
SDS	2	5.03094 × 10^–6^	3.0297040 × 10^21^	0.3778214	20,000	7556.43	5 × 10^–10^
OTG	1	4.85054 × 10^–6^	2.9210641 × 10^21^	4.12739	10,000	41273.9	5 × 10^–9^

a Note that large numbers of significant figures are required to avoid round off errors when converting between molecules and moles.

b and Γ_∞_ determined by fitting macroscopic data to the Langmuir isotherm, *n* set to 1 or 2 for the isotherm fit.

c [Site]_max_ for a droplet with 25 μm radius.

d Allowed to vary during kinetic modeling with starting guesses of *D*_apparent_ = 5 × 10^–10^ m^2^/s and *k*_solv_ = 100 s^–1^.

e *k*_desolv_ constrained by *K* and *k*_solv_.

#### Microscopic Droplet Surface Tension Measurements

**Figure 1:**
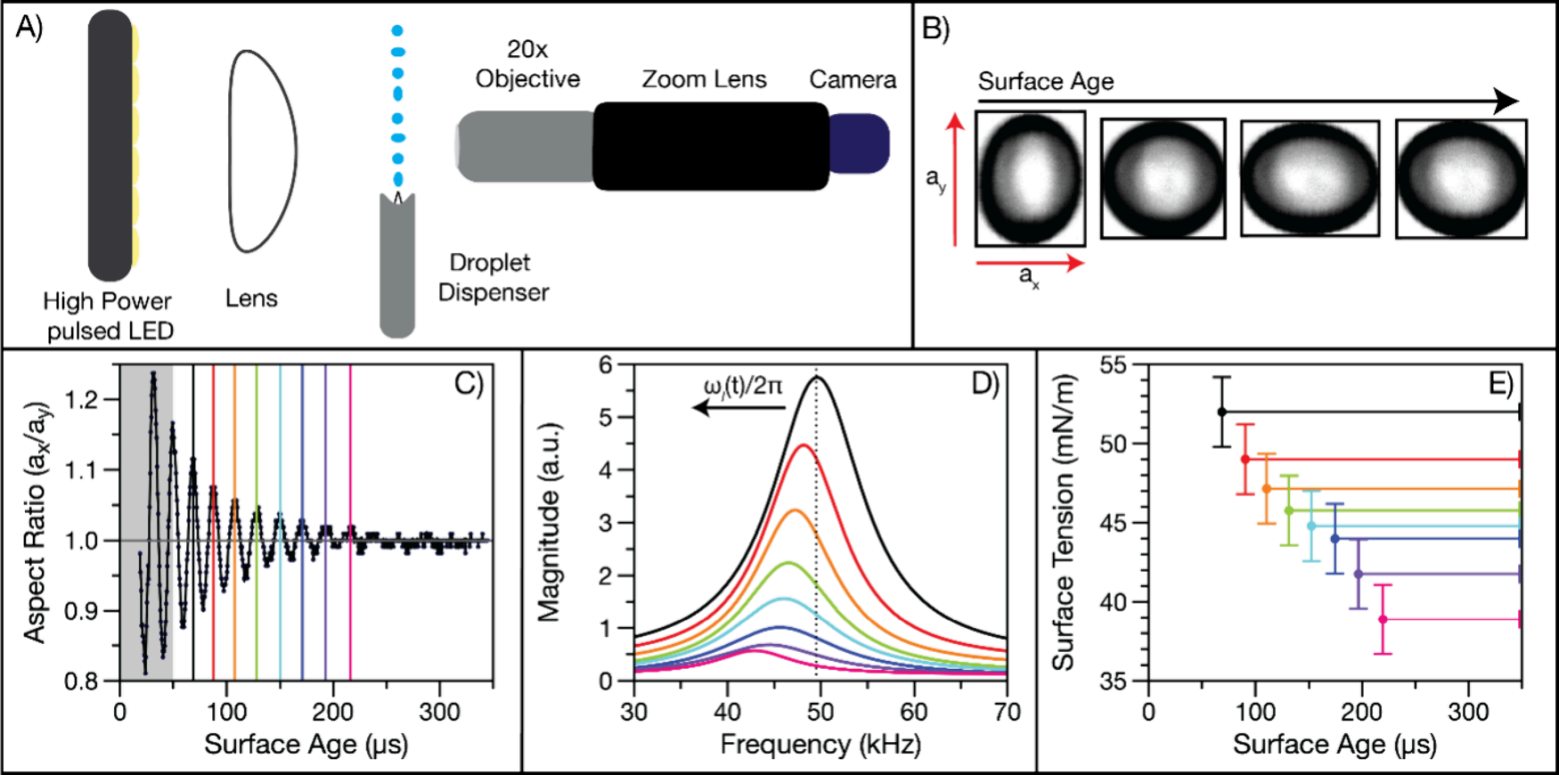
Experimental workflow. (A) Oscillating droplet experimental setup: a train of identical droplets is ejected from a droplet dispenser and stroboscopically imaged by stepping up the time between droplet generation and image capture. (B) Images of droplets at different times after generation. The aspect ratio, *a*_*x*_/*a*_*y*_, changes as the droplets oscillate. (C) Aspect ratio as a function of surface age built up from hundreds of droplet images. The shortest time points (gray-shaded region) are removed to eliminate nonlinear effects resulting from aspect ratios greater than 1.2 and influences from higher-order surface modes. The aspect ratio trace is spliced at each peak and a fast Fourier transform (FFT) is taken from each splice to the end of the trace. (D) Lorentzian fits to determine oscillation frequency resulting from the FFT from each splice. Vertical dotted line shows the frequency at the earliest time point to guide the eye. (E) Retrieved surface tension for each splice showing surface tension dynamics as the droplet surface ages. *y*-error bars represent measurement uncertainty from the uncertainty in the droplet radius; *x*-error bars show the time frame that is included in the FFT. In panels C–E, droplets contain OTG and the colors are consistent such that the surface tension and oscillation frequency in panels D and E in any color result from splicing the data from the same colored line in panel C.

The dynamic surface tension of microscopic (14–25 μm radius) aqueous droplets containing surfactants was measured over several tens to hundreds of microseconds after droplet generation using a previously described stroboscopic imaging approach.^57^ A diagram of the optical setup is shown in Figure 1A. A train of monodisperse droplets is generated using a piezoelectric droplet dispenser (MicroFab MJ-ABP-01) with a 30 μm-diameter orifice. Droplets are ejected at a frequency that is typically 10–20 Hz. Droplet size can be tuned by changing the amplitude and duration of an electrical pulse applied to the dispenser. A given set of dispenser parameters allows generation of a highly stable and reproducible droplet train.^58^

Ejection of the droplets from the dispenser excites fundamental surface oscillations on the droplet. The frequency of these surface oscillations allows quantification of the droplet surface tension (σ) through eq 3(59):
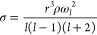
3where *r* is the droplet radius, ρ is the droplet density, and ω_*l*_ is the angular oscillation frequency of surface mode order *l*.

The droplet oscillatory modes are recorded with a stroboscopic imaging setup consisting of a high-power LED (GSVITEC MULTILED QX) light source pulsed at 500 ns, a microscope objective (Mitutoyo, Plan APO Infinity corrected long working distance, 20×), a zoom lens (Navitar, 1-80100D), and a camera (JAI GO-2400M-USB). The delay time between droplet generation and image capture was stepped up in 500 ns increments, allowing time-dependent measurements of a droplet’s shape (Figure 1B).

Custom LABVIEW software automatically identifies the droplet in the 8-bit grayscale image and calculates the aspect ratio (*a*_*x*_/*a*_*y*_). Once the surface modes damp, the droplets return to a spherical shape and the aspect ratio relaxes to *a*_*x*_/*a*_*y*_ = 1. The droplet diameter is retrieved as the average of 100 measurements of droplet size once *a*_*x*_/*a*_*y*_ = 1. The time scale of the measurement is extremely short (a few hundred microseconds after pinch-off, defined here as *t* = 0), while the time scale required for solvent evaporation is much longer, meaning that solvent evaporation from the droplet during the experiment is minimal and therefore can be neglected. For example, under typical laboratory conditions (59% RH and 298 K), a water droplet initially 25 μm radius would shrink <2% in radius due to evaporation over 100 ms.^60^

The *l* = 2 surface mode order is used to determine surface tension since it oscillates the longest before damping. Data in which the droplet aspect ratio reached >1.2 were removed for two reasons. First, oscillation amplitudes >10% of the droplet radius can lead to nonlinear effects on the droplet oscillation frequency.^61^ In some instances, nonlinear effects have been observed in oscillating droplets at smaller amplitude oscillations, leading to recommendations for the use of the droplet oscillation approach under conditions where the Ohnesorge number (Oh) is ≤0.04.^62^ For our measurements, Oh ranged from 0.017 to 0.035. Second, at the shortest time after excitation of the oscillations, higher-order modes may also contribute to the retrieved droplet aspect ratio. By removing early data points, contributions from higher-order modes, which damp more quickly than the fundamental *l* = 2 mode,^61^ are eliminated. Depending on droplet size, approximately 50–150 μs could be removed from the beginning of the trace (gray-shaded region in Figure 1C) before proceeding to determine surface tension. Additional factors that have been found to skew the retrieved frequency of oscillating droplets, such as asymmetric oscillations,^62^ are not observed in our measurements.

A fast Fourier transform (FFT) of the aspect ratio trace is used to determine the oscillation frequency, ω_*l*_, of the *l* = 2 surface mode. To determine how the surface concentration, and thus the surface tension, changes with time, we splice the aspect ratio trace at each peak and perform an FFT using all data points to longer times (i.e., to the right in Figure 1C). The resulting power spectrum is fit with a Lorentzian function to retrieve the oscillation frequency (Figure 1D). An uncertainty on oscillation frequency is quantified from the goodness of this fit. If the uncertainty on the oscillation frequency results in an uncertainty in surface tension >2 mN/m, that splice is ignored. This process is repeated until three oscillation periods are left in the trace. Analyzing fewer than three oscillations provides insufficient information for retrieving the oscillation frequency.

From the time-resolved oscillation frequencies, droplet surface tensions are calculated for each splice using eq 3. The radius is taken as half the diameter retrieved from the droplet images after the surface modes have damped (*a*_*x*_/*a*_*y*_ = 1). Droplet density is assumed equivalent to that of the solvent, water (998.23 kg/m^3^), given the surfactant concentrations used in this study are all ≤50 mM. Macroscopic solution densities, obtained using a density meter (Density2Go, METTLER TOLEDO), confirm that the solution density is equivalent to that of water. Data sets at each surfactant concentration were collected for droplets across a range of sizes. All presented droplet surface tension data are averaged into time bins from data across >3 droplet train experiments. If the standard deviation in a time bin is smaller than the typical error introduced from uncertainty in the droplet radius (2.2 mN/m), it is increased to this value. An example of the resulting dynamic surface tension is shown in Figure 1E.

### Kinetic Modeling of Surfactant Partitioning in Microscopic Droplets

We use a model built in Kinetiscope^24,63^ to describe the kinetics of surfactant transport to the interface for 14–25 μm radius aqueous droplets containing surfactant, either SDS or OTG. Models built in Kinetiscope have previously been used to explain the kinetics of droplet evaporation, the multiphase chemistry of aerosols, and reactions in emulsions.^24,64−71^ The model for surfactant transport to the interface is based on the Langmuir equation (eq 1), in which it is assumed that the partitioning of the nonvolatile surfactant to the interface is governed by the equilibrium constant (*K*_eq_^surf^, eq 4), which is the ratio of the desolvation (desolv) and solvation (solv) rate constants.
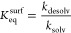
4

We adopt a rectangular prism simulation geometry (1 nm × 1 nm × *r*/3) developed by Houle and co-workers^70^ but expanded to include three compartments (a bulk compartment, an adsorption compartment, and the surface) to represent the droplet (Figure S2). We include an adsorption compartment between the droplet bulk and the surface to correctly model subsurface concentrations that are not strictly governed by diffusion. The surfactant concentration in the adsorption compartment is determined by the competing kinetics of diffusion from the bulk and adsorption to the surface.^33^

The length scales of the three-compartment simulation geometry are unique to each surfactant and each concentration. First, the adsorption compartment is set to a length of [surf_(ads)_]_max_/[surf_(bulk)_]_0_ (i.e., the ratio of the maximum surface concentration at equilibrium to the bulk concentration at *t* = 0). Second, the length of the bulk compartment is set to , where *r* is the droplet radius, so that the surface-area-to-volume ratio of the droplet is maintained (with the length of the adsorption + bulk compartments = ). Finally, the surface compartment is set to a thickness, δ, of 1 nm, resulting in a total number of surface sites, [sites]= . A surface thickness of 1 nm is approximately the thickness of three water molecules and is consistent with density and solvation energy profiles from molecular dynamics simulations.^1,72,73^ From the Langmuir equation, the concentration of surfactant adsorbed at the interface at equilibrium is
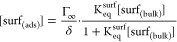
5

At time *t* = 0, both the droplet bulk and the adsorption compartment have concentrations equal to the total surfactant concentration and surfactant is allowed to undergo bidirectional gradient diffusion between the compartments throughout the simulation. The surfactant in the adsorption compartment (Surfactant_(ads compartment)_) is coupled with the surface compartment through diffusion. Once present in the surface compartment, the surfactant undergoes adsorption to and from the interface by the rate constants *k*_desolv_ and *k*_solv_. The elementary steps used in the surface compartment of the simulations are
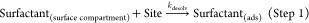

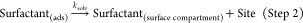


An expression for size-dependent surface tension is obtained by solving a set of equations relating surfactant desolvation and solvation at equilibrium.^23^ This is done by solving explicitly for surface coverage, Θ, previously described by the Langmuir equation of state in (eq 2), which is used to compute surface tension, σ (eq 1). For a droplet of radius *r*, surface coverage can no longer be described by the Langmuir equation due to simultaneous depletion of the bulk concentration [surf_(bulk)_], a consequence of the large surface-area-to-volume ratio of microdroplets. Surface coverage, Θ, can be more generally defined
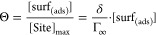
6where eq 6 simply expresses Θ as the fraction of occupied surface sites, the maximum being equal to . To solve for [surf_(ads)_], we relate the rates of surfactant desolvation and solvation. At equilibrium, the desolvation and solvation rates are equal. We use this equilibrium description, as well as the initial bulk concentration [surf _(bulk)_]_0_ to construct the set of equations:
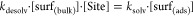
7
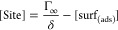
8
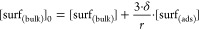
9Above, eq 8 conserves the total site number and eq 9 conserves the total number of surfactant molecules. The concentration of adsorbed surfactant [surf_(ads)_] in eq 9 must be weighted by *r*/3 to account for the surface-to-volume ratio of the droplet. Solving eqs 7–9^23^ provides the equilibrated [surf_(ads)_]:
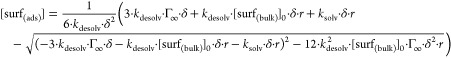
10

The model output, the concentration of surfactant adsorbed at the interface over time ([surf_(ads)_]), is then used with eqs 6 and 1 to determine the surface tension. In some cases, the calculated surface tension is lower than the minimum surface tension determined from macroscopic measurements. This is due to the minimum surface tension occurring before the fractional surface coverage reaches one using experimental data to fit eqs 1 and 2 (Figure S3). This behavior has been previously observed.^74^ When the retrieved surface tension is lower than the minimum surface tension determined from macroscopic equilibrium measurements, it is replaced with a limiting value (38.7 mN/m for SDS and 30.0 mN/m for OTG). It is not expected that the minimum surface tension in micrometer- or larger-sized droplets would be lower than the minimum surface tension measured for macroscopic solutions. When the droplet size is sufficiently large (∼100 μm here), the kinetic model returns the Langmuir isotherm fit (Figure S1).

To run the time-dependent model, we require input for the diffusion coefficient, *D*, of the surfactant in water as well as the rate constants *k*_desolv_ and *k*_solv_. The equilibrium rate constant, *K*_eq_^surf^, is quantified by fitting macroscopic surface tension measurements of aqueous SDS and OTG to the Langmuir isotherm (Table 1 and Figure S1). Here, we have used K_eq_^surf^ determined from such fits to constrain the ratio of *k*_desolv_ to *k*_solv_, and we vary *k*_solv_. We initially set *k*_solv_ to 100 s^–1^, a value consistent with previous literature on alcohols and small dicarboxylic acids.^75^ These rate constants are difficult to measure, and there are limited and inconsistent observations for larger surfactants. The diffusion coefficient for surfactants in aqueous solution is generally agreed to be on the order of 5 × 10^–10^ m^2^/s in experimental measurements and molecular dynamics simulations,^76−79^ which is used as the initial guess for the kinetic model. For each surfactant, we chose one concentration as a test case (6 mM SDS or 10 mM OTG). These concentrations were selected because a clear change in surface tension with surface age was observed over the experiment and they do not contain any surface ages shorter than 40 μs, which are expected to be systematically reduced due to the surface tension retrieval method which uses the full aspect ratio trace for the shortest surface ages. We increase *k*_solv_ (maintaining *K*_eq_^surf^ to the value determined from the macroscopic measurements) until the model begins to overlap with the test experimental data set. In the case of OTG, a limit was reached where increasing *k*_solv_ no longer shifted the model output to earlier times before the data and model predictions overlapped. We then increased *D* until agreement was observed between the model predictions and data. Once *D* and *k*_solv_ were found for data-model agreement in the test case, these parameters were used to simulate the surface tension dynamics for all other concentrations of that surfactant. Fit parameters for each surfactant are provided in Table 1.

## Results and Discussion

### Measurements of Droplet Surface Tension as a Function of Surface Age

The dynamic surface tensions of picoliter droplets containing different concentrations of either SDS or OTG surfactants were monitored microseconds after surface formation. Figure 1C–E provides an example of the dynamic behavior observed for droplets containing the surfactant OTG, whereas Figure S4 shows data for pure water droplets^57^ processed in the same manner. Figure S4 demonstrates that, for pure water droplets, splicing the oscillation trace and performing FFTs over different time frames does not alter the retrieved oscillation frequency (Figure S4A). The retrieved droplet surface tensions consistently cluster around the expected value for water, 72.8 mN/m, regardless of the droplet’s surface age (Figure S4B). By contrast, for the aqueous OTG droplet in Figure 1, the oscillation frequency systematically shifts to lower values at longer droplet surface ages (Figure 1D), leading to the retrieved droplet surface tension decreasing toward the expected equilibrium value at longer droplet surface ages (Figure 1E). The difference in the general magnitude of oscillation frequency between Figure 1D (∼45–50 kHz) and Figure S4A (∼33 kHz) is a result of the difference in radius between the two droplets (∼22 μm for the OTG droplet in Figure 1 and ∼24 μm for the water droplet in Figure S4). The reduction in droplet surface tension with increasing surface age for the aqueous OTG droplet but not for the pure water droplet shows that the time-dependent change in surface tension is due to the presence of the surfactant, which partitions to the droplet–air interface on time scales spanning several hundreds of microseconds in droplets with radii in the 10s of micron range (11–65 pL).

**Figure 2:**
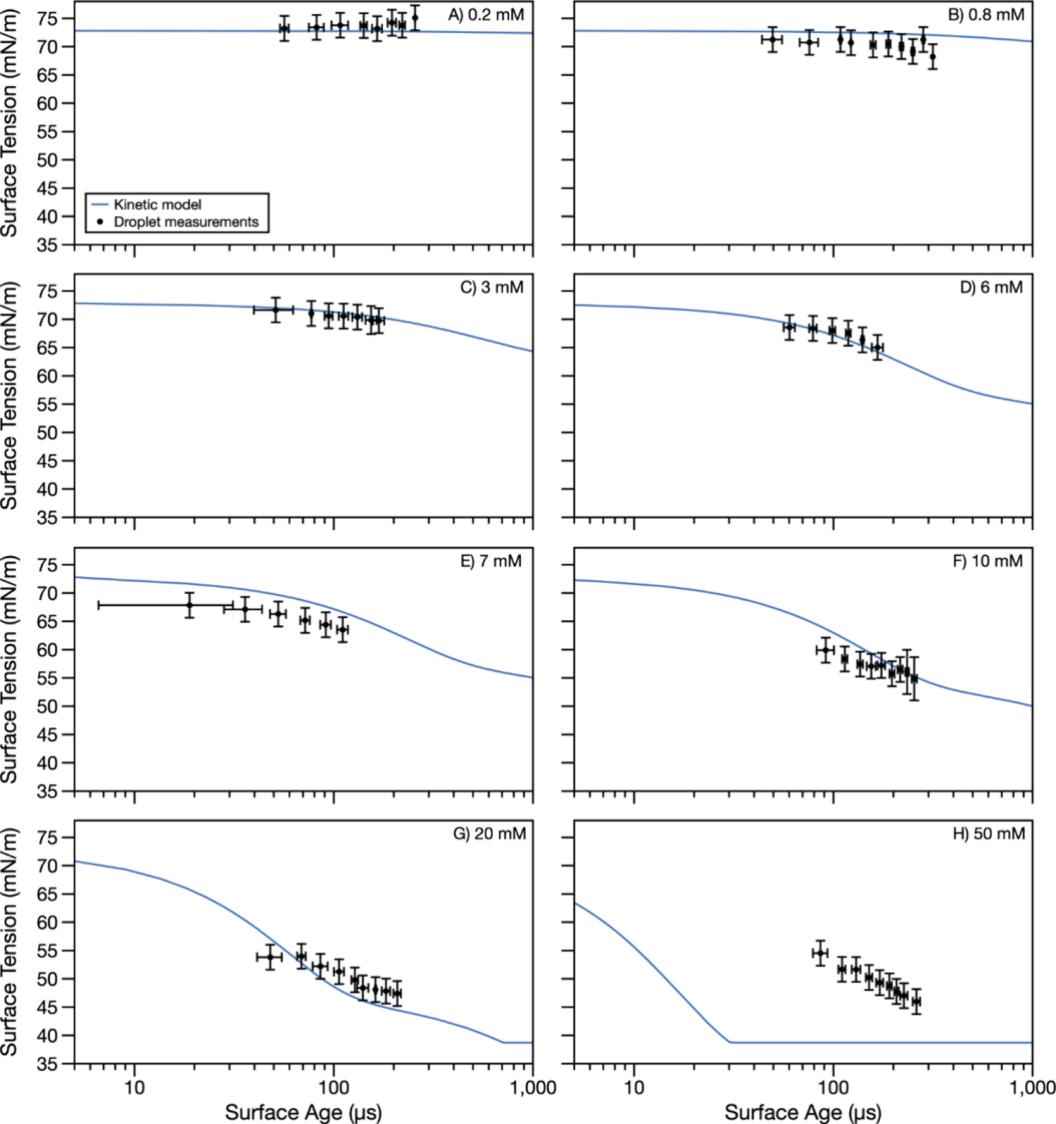
Dynamic surface tension of SDS droplets with concentrations of (A) 0.2, (B) 0.8, (C) 3, (D) 6, (E) 7, (F) 10, (G) 20, and (H) 50 mM. Droplet measurement data points represent an average of multiple experiments with droplet radii in the 14–25 μm range, binned in time. Error bars in the *x*-direction represent the standard deviation of surface age in a bin. Error bars in the *y*-direction are the larger of the standard deviation of surface tension in a bin or 2.2 mN/m (the calculated measurement uncertainty from uncertainty in the droplet radius). Dynamic surface tension predictions from the kinetic model using parameters in Table 1 are shown in blue.

The time-dependence of surfactant partitioning to the droplet–air interface was further investigated for two surfactants, SDS and OTG, at a range of surfactant concentrations. Figures 2 (SDS) and 3 (OTG) show dynamic surface tension measurements for 14–25 μm radius droplets spanning a range of surfactant concentrations. From the experimental results, it is obvious that as the surfactant concentration in the droplets increases, observed droplet surface tensions decrease, with clear time dependencies that are unique to each specific measurement. At low surfactant concentrations, droplet surface tensions are initially closer to 72.8 mN/m (the solvent surface tension), whereas at higher surfactant concentrations, droplet surface tensions trend toward the expected equilibrium value. Within each measurement, the time-dependent surface tension generally trends lower at longer droplet surface ages, though the magnitude of the time-dependent change in surface tension depends on the surfactant identity and concentration for that experiment. Overall, the observed dynamics are different for the two surfactants, indicating they arise from the unique interfacial adsorption properties for each surfactant, even though it takes a similar amount of each surfactant to reach the minimum equilibrium surface tension (Figure S1, 8.7 and 9.1 mM for SDS and OTG, respectively).

**Figure 3:**
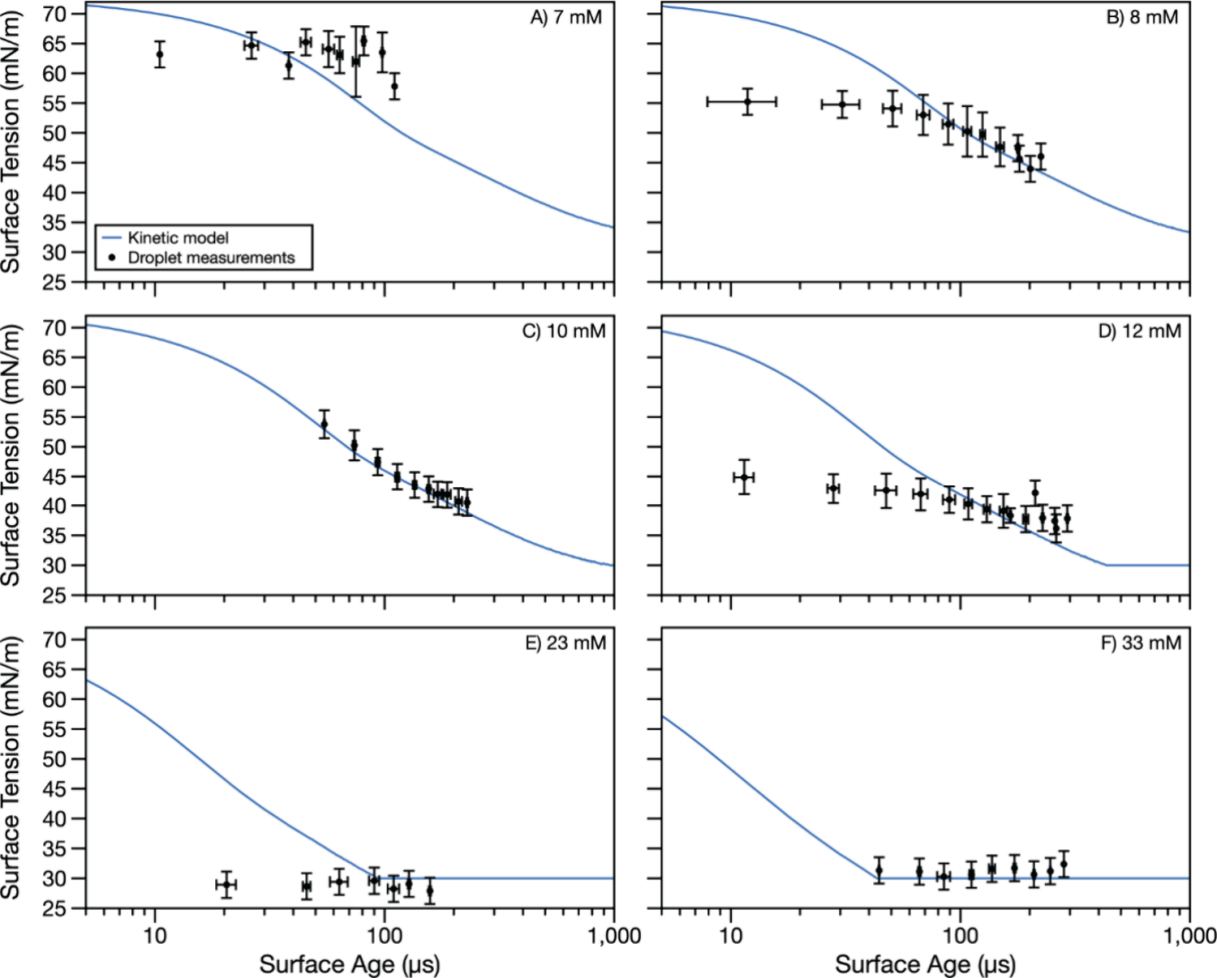
Dynamic surface tension of OTG droplets with concentrations of (A) 7, (B) 8, (C), 10, (D) 12, (E) 23, and (F) 33 mM. Droplet measurement data points represent an average of multiple experiments with droplet radii in the 14–25 μm range, binned in time. Error bars in the *x*-direction represent the standard deviation of surface age in a bin. Error bars in the *y*-direction are the larger of the standard deviation of surface tension in a bin or 2.2 mN/m (the calculated measurement uncertainty from uncertainty in the droplet radius). Dynamic surface tension predictions from the kinetic model using parameters in Table 1 are shown in blue.

### Kinetic Modeling of the Dynamic Surface Tension of Aqueous Droplets

To interpret the experimental data, the measurements were fit to a kinetic model using the protocol described in the Experimental Section. Importantly, by incorporating the adsorption compartment and maintaining the surface-area-to-volume ratio, this model accounts for bulk depletion effects inherent to aerosol droplets. Model fits (assuming a droplet radius of 25 μm) are represented by the solid lines in Figures 2 and 3. For SDS (Figure 2), the model was initially fit only to experimental measurements made at 6 mM concentration. The best-fit parameters were then applied to experimental data collected across all eight studied SDS concentrations. Similarly, for OTG (Figure 3), the model was initially fit only to experimental measurements made at 10 mM concentration, with the resulting best-fit parameters applied to the experimental data collected across all six studied OTG concentrations.

A single set of surfactant parameters is sufficient to represent the majority of the experimental data (see Table 1). For SDS, the *D*_apparent_ and *k*_solv_ values required for overlap between the experimental data and model are 5 × 10^–10^ m^2^/s and 20,000 s^–1^, respectively; for OTG, these values are 5 × 10^–9^ m^2^/s and 10,000 s^–1^. The *D*_apparent_ value for SDS is of similar magnitude to that expected for dilute surfactants based on macroscopic measurements.^76−79^ However, *D*_apparent_ for OTG is about an order of magnitude higher than that expected based on macroscopic measurements for dilute surfactants and predictions using the Stokes–Einstein equation.^80,81^*k*_solv_ is much larger than measurements for small carboxylic acids.^75^ We are unable to compare the retrieved rate constants to literature values due to a dearth of such measurements.

For most of the data sets, the surface tension predicted with the kinetic model falls through the experimental data, and there is often good agreement between the model and the experimental data both in terms of the magnitude of surface tension and the shape of the dynamics. For SDS, the model accurately predicts time-dependent droplet surface tensions across nearly all surfactant concentrations. The model only does a poor job at the highest concentration investigated (50 mM, Figure 2H). Interestingly, once the surfactant concentration reaches 20 mM (Figure 2G), increasing the surfactant concentration does not greatly affect the experimentally observed dynamics. For the 20 and 50 mM data sets, the measured time-dependent surface tensions are very similar, whereas the model predicts large differences. Because the kinetic model is based on the Langmuir isotherm, it assumes that (1) every adsorption site at the interface is equivalent, (2) the probability of adsorption to an empty site is independent of the occupancy of neighboring sites, and (3) there are no interactions or intermolecular forces between surfactant molecules at the interface.^82^ It is possible that these assumptions hold true for SDS (an ionic surfactant) at low surface coverage but no longer hold as the droplet surface becomes more saturated with SDS molecules. In macroscopic solutions, *k*_solv_ and *k*_desolv_ have sometimes been found to be functions of surfactant concentration or experimental parameters,^83−86^ suggesting that these common assumptions may not necessarily always hold true.

For droplets containing the surfactant OTG (Figure 3), the kinetic model largely aligns with the experimental measurements. However, for half of the concentrations investigated, the model predicts a higher surface tension at the shortest surface age than measured by the experiment (i.e., Figure 3B,D,E). The disagreement between experimentally determined surface tension and the model is prevalent for surface ages shorter than 40 μs. The disagreement between model and measurement may arise because the data analysis approach systematically reduces the retrieved surface tension at shorter surface ages due to incorporation of oscillations at later times into the FFT (see the Experimental Section and Figure 1). Nonetheless, at longer surface ages in Figure 3B,D,E, the model exhibits close agreement with the experimental measurements. It is notable that the measurements and model agree on the minimum (equilibrium) surface tension for OTG.

### Model Sensitivity Analysis

We also explored the sensitivity of model parameters to matching experimental measurements. The sensitivity for the parameters *D*, *k*_solv_, and *k*_desolv_ was explored for the specific cases of 10 mM SDS droplets (Figure S5) and 10 mM OTG droplets (Figure S6). For SDS, *D* was varied between the approximate limits of reported diffusion coefficients for aqueous surfactants (1 × 10^–10^ to 9 × 10^–10^ m^2^/s) and *k*_solv_ was increased and decreased by a factor of 2, 5, and 10. For OTG, *D* was changed toward the expected literature value (5 × 10^–10^ m^2^/s). For both systems, the value for *k*_desolv_ was altered to maintain the experimentally determined ratio *K*_eq_^surf^ (reported in Table 1). In Figures S5 and S6, the gray line shows the initial guess, *D*_lit_ = 5 × 10^–10^ m^2^/s, and *k*_solv, lit_ = 100 s^–1^.

For both SDS and OTG, the kinetic model is more sensitive to changes in the diffusion coefficient than to changes in the rate constants. The calculated critical radii () for mass transport^23^ using the determined fit parameters in Table 1 are 44 and 61 nm for SDS and OTG, respectively. This critical radius describes the radius of a droplet above which diffusion would limit surfactant transport to the interface. Since the droplets investigated here are larger than this critical radius (*r* >14 μm), mass transport to the interface is limited by diffusion.^23^ Increasing *k*_solv_, even by an order of magnitude, barely changes the surface tension prediction. Decreasing *k*_solv_ has a greater effect, slowing down the predicted partitioning dynamics. These results suggest retrieving an accurate value for *k*_solv_ by fitting these experimental data may be challenging, although a lower limit on its magnitude can be quantified. In contrast, the kinetic model is highly sensitive to the diffusion coefficient and a two-compartment model (surface and bulk) does not well describe the dynamics observed in the experimental data. The requirement of the (third) adsorption compartment indicates that the surfactant mass transport to the interface is diffusion-limited. For SDS, increasing *D* to 9 × 10^–10^ m^2^/s shifts the predicted surface tension dynamics faster, reducing the predicted surface tension in our observation window by about 5 mN/m. Decreasing *D* to 1 × 10^–10^ m^2^/s slows the dynamics, with predicted surface tension only lowering to about 65 mN/m in 300 μs. For OTG, reducing *D* toward the expected (literature) diffusion coefficient dramatically slows the predicted surface tension dynamics, leading to larger disagreements with the measurements, with *D* = 1 × 10^–9^ m^2^/s overpredicting the surface tension by nearly 15 mN/m and *D* = 5 × 10^–10^ m^2^/s overpredicting by about 25 mN/m at 250 μs.

The apparent diffusion coefficient required to bring the OTG model and droplet data into agreement is an order of magnitude faster than the literature value. The high diffusion coefficient required may be due to systematic uncertainties in the start time for surfactant partitioning and our simplified kinetic model. For example, in our analysis, time *t* = 0 is defined as the time when the droplet pinches off from the liquid in the dispenser. However, some surfactant may also partition to the meniscus at the dispenser tip between droplet ejection events, though a simulation assuming some initial surfactant partitioning to the droplet meniscus does not explain the higher required *D* (Figure S7). Moreover, shifting the data along the time axis does not improve the agreement with model predictions. Additionally, our simulations assume a spherical geometry, but at times directly after pinch-off, the aspect ratio can be >1.2. While these initial oscillations are not included in the analysis (see the Experimental Section), a large aspect ratio could reduce the radial distance for diffusion and potentially lead to faster apparent diffusion than expected for a spherical droplet of the same volume. However, the oscillation time scales in these experiments are on the order of tens of microseconds, whereas the surfactant partitioning time scales are on the order of hundreds to thousands of microseconds. The kinetic model is weakly sensitive to the droplet radius across the size range investigated in this study (14 and 25 μm radius, Figures S8 and S9), with droplet size potentially leading to a maximum of 5 mN/m differences across the measured droplet size range. This weak dependence on droplet size is consistent with measurements (Figure S10). Finally, the droplet generation process could induce convection currents within the droplet, which may alter the apparent diffusion constant from the literature value.^87^ Although we observe little size dependence for the dynamics of surfactant partitioning in droplets of radius 14–25 μm, we do expect partitioning dynamics and time scales to change as droplet radius is decreased into the submicron size range.^33,51^ The kinetic model we describe here accounts for the surface-area-to-volume ratio of the droplet and can be used to predict the partitioning in smaller volumes once kinetic parameters are known.

### Predictions of Time- and Concentration-Dependent Droplet Surface Coverage

**Figure 4:**
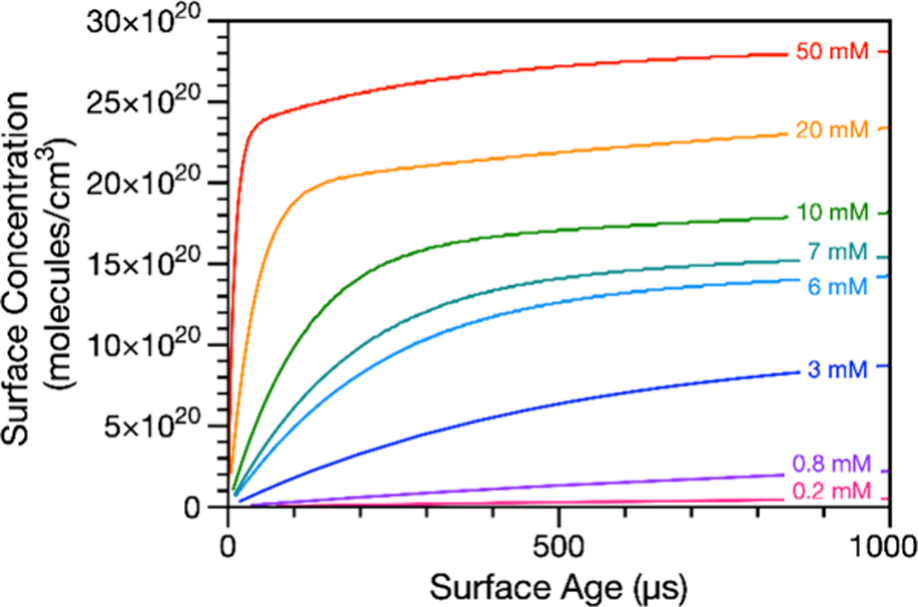
Predicted surface concentration of SDS adsorbed in the δ = 1 nm-thick surface of a 25 μm radius droplet containing 0.2–50 mM total SDS for the first 1000 μs after droplet generation.

In Figure 4, the kinetic model is used to predict surfactant surface concentrations in a 25 μm radius aqueous SDS droplet as a function of time (up to 1 ms surface age) and SDS concentration (0.2–50 mM). The surface thickness is assumed to be 1 nm. Regardless of the initial bulk surfactant concentration, the surface concentration at *t* = 0 is set to 0 molecules/cm^3^. Shortly after formation of the droplet (<100 μs), SDS begins to populate the interface. For droplets with a high bulk SDS concentration, the surface concentration increases rapidly over several 10s of microseconds. However, for droplets with lower initial bulk SDS concentrations, the time required for surfactant to populate the interface increases significantly. For 3 mM SDS, the kinetic modeling shows that the surface concentration quickly increases to 8.74 × 10^20^ molecules/cm^3^ (<60% of the equilibrium surface concentration, 1.52 × 10^21^ molecules/cm^3^) in 1 ms, followed by a much slower increase in surface coverage. In simulations allowed to run for longer times, about 40 ms is required for the 25 μm radius droplet containing 3 mM SDS to reach its equilibrium surface concentration (Figure S11). Conversely, a 50 mM SDS droplet approaches its maximum surface concentration (3.03 × 10^21^ molecules/cm^3^) in the 1 ms time frame of the simulation in Figure 4. Droplets with lower bulk concentrations (but still sufficiently high bulk concentrations to have the same equilibrium surface coverage, i.e., 10 and 20 mM) take much longer to reach their equilibrium surface concentrations. We note that at these concentrations and for the 25 μm radius droplets accessible with our measurement technique, the droplet surface tension measurements and model are in good agreement (Figure 2F,G). The simulations shown in Figure 4 highlight the importance of considering surfactant partitioning effects over the short (micro- to millisecond) time scales during which chemistry is expected to occur in microcompartments like aerosol droplets.^49,88,89^ Ignoring these time scales could lead to incorrect assumptions about reaction dynamics and efficiencies, which may have practical impacts in different application domains.

## Conclusions

The surface tensions of microscopic ∼20 μm radius (∼10–60 pL) droplets were quantified over time scales of tens to hundreds of microseconds after generation using a stroboscopic imaging approach. Different surfactants exhibited unique partitioning dynamics that depended on their kinetic properties. The experimental data at one surfactant concentration were fit to a kinetic model that accounts for the high surface-area-to-volume ratio environment of microdroplets. The best-fit parameters were then applied to droplet measurements for that surfactant at several different concentrations. In most cases, the kinetic model predictions matched experimental observations. For the specific droplet sizes and concentrations examined here, the model was very sensitive to the diffusion constant but less sensitive to the solvation and desolvation rate constants (only permitting quantification of lower limits for these parameters). The model demonstrates that for a typical surfactant (SDS), time scales on the order of many tens of milliseconds may be required for the droplet to reach its equilibrium surface composition, a time scale similar to or even longer than the reaction time scales observed during accelerated chemistry in microdroplets. Based on these time scales, chemistry at the droplet–air interface in freshly generated microdroplets may proceed under nonequilibrium concentrations.

Mechanistic understanding of dynamic droplet surface properties is essential to resolve both how the surface tension of aerosols and droplets evolves over time and how chemical reaction dynamics can be altered in microdroplets. Since the significance of interfacial chemistry is enhanced in microcompartments and because many molecules that undergo chemical reactions (in contexts that include atmospheric science and chemical synthesis) are surface-active to varying degrees, these partitioning time scales should be considered when attempting to explain observations of accelerated chemistry in microcompartments.^23^ Measurements on the model surfactant systems studied here help to validate the kinetic modeling approach, ensuring it can rationalize multiple independent experimental observations in a self-consistent manner and providing confidence in the application of the model to systems not containing surfactants. Although this contribution provides information about how surfactants partition to the interface over microsecond time scales, it also highlights areas for future work. First, work should aim to explore the dynamics of interfacial structure, building on recent surface spectroscopic advances in this area.^42−45^ Second, the complex relationship between surfactant concentration and aerosol size, leading to bulk concentration depletion, and its impact on partitioning time scales should be explored. Moreover, future efforts should focus on expanding the time scales over which partitioning dynamics can be investigated and exploring more complex systems, such as surfactant–surfactant or surfactant–cosolute mixtures, where additional molecules can alter surface partitioning.

## Data Availability

All data underlying the figures are available through the University of Bristol data repository, data.bris, at https://doi.org/10.5523/bris.4wbdqu6bt702wsortm322e4u.

## Supporting Information Available

Macroscopic surface tension measurements and isotherms, visualization of simulation geometry, oscillating droplet results for pure water droplets, sensitivity tests to input model parameters (kinetic constants, diffusion coefficients, droplet radius, and initial surface coverage), experimental data comparing droplets of different sizes accessible with our techniques, and kinetic simulation results for up to 50 ms after surface formation (PDF)
