# Supplementary material for: Surfactant Partitioning Dynamics in Freshly Generated Aerosol Droplets
Source: J Am Chem Soc. 2024 Jun 1;146(23):16028–38. doi: 10.1021/jacs.4c03041 (PMC11177314; doi:10.1021/jacs.4c03041)
Supplement: Supplementary file 1 — ja4c03041_si_001.pdf [file ja4c03041_si_001.pdf]

Supplementary Information for:
Surfactant Partitioning Dynamics in Freshly Generated Aerosol Droplets

Submitted to *JACS*

Alison Bain,[1,2] Lara Lalemi,[1] Nathan Croll Dawes,[1] Rachael E. H. Miles,[1] Alexander M. Prophet,[3,4] Kevin R. Wilson,[4] and Bryan R. Bzdek[1*]

[1] School of Chemistry, University of Bristol, Cantock's Close, Bristol UK BS8 1TS

[2] Department of Chemistry, Oregon State University, Corvallis, OR, 97331

[3] Department of Chemistry, University of California, Berkeley, CA, 94720

[4] Chemical Sciences Division, Lawrence Berkeley National Laboratory, Berkeley, CA, 94720

*b.bzdek@bristol.ac.uk

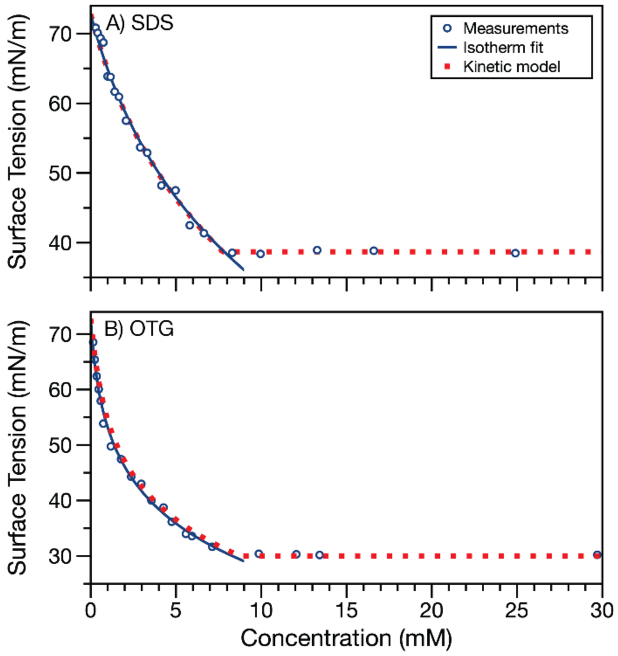

Figure S1: Macroscopic surface tension data (open blue points) and Langmuir isotherm fit (solid blue lines). Red dotted line shows kinetic model predictions for a 100 μm radius droplet, which is large enough to behave like a macroscopic solution. A) SDS B) OTG[21].

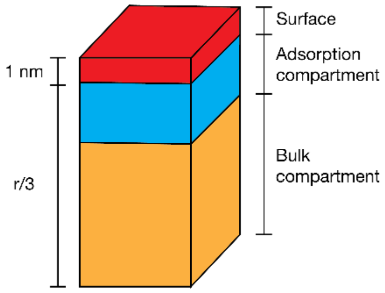

Figure S2: Visual description of the geometry for the three-compartment Kinetiscope simulation, which consists of a bulk, adsorption, and surface compartment.

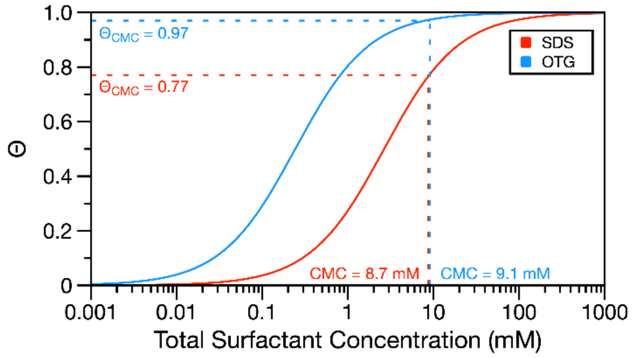

Figure S3: Fractional surface coverage, Θ, as a function of total surfactant concentration for SDS (red) and OTG (blue). Vertical dashed lines show the CMC determined from macroscopic equilibrium surface tension measurements and horizontal dashed lines indicate the corresponding Θ from Eq. 2.

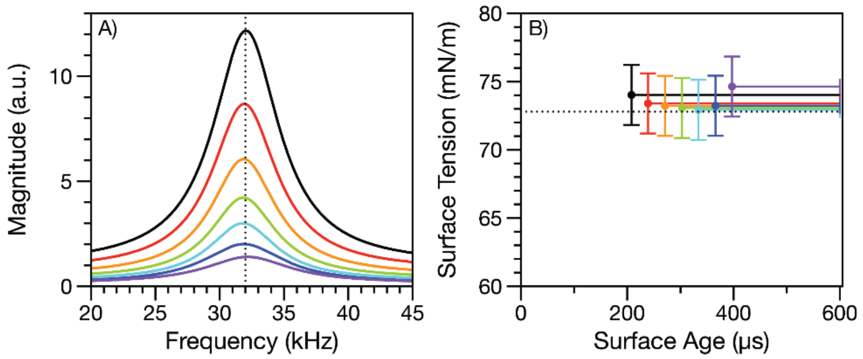

Figure S4: Dynamic A) oscillation frequency and B) surface tension of a train of water droplets. Water droplet aspect ratio in time from Miles et al.[42] *was analyzed following the same procedure as the OTG containing droplets shown in Fig. 1 of the main text. The dotted vertical line in panel A shows the frequency at the earliest time point to guide the eye (black curve), and the dotted horizontal line in panel B shows the expected surface tension of water, 72.8 mN/m.*

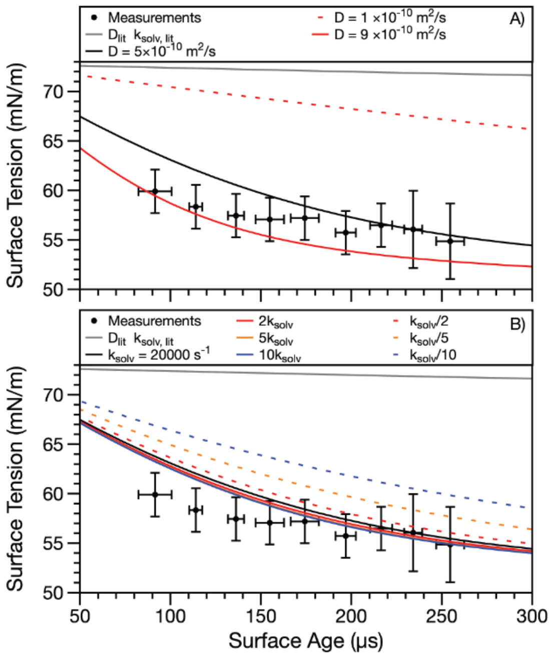

Figure S5: Sensitivity of model to input diffusion coefficient (D) and rate constants k_{solv} and k_{desolv} for 10 mM SDS. Black lines simulated with the parameters in Table 1. A) k_{solv} is held constant at 20,000 s^{-1} and D is shown at 1 and 9×10^{-10} m^2/s, approximate limiting cases to literature surfactant diffusion coefficients. B) D is held constant at 5×10^{-10} m^2/s and k_{solv} is varied by a factor of 2, 5, and 10. (k_{desolv} is also varied to keep the ratio K_{eq}^{surf} constant.) The grey line near the top of each panel shows the dynamics calculated with literature values for a generic surfactant, $D_{lit} = 5\times10^{-10}$ m^2/s and $k_{solv,\ lit} = 100$ s^{-1}.

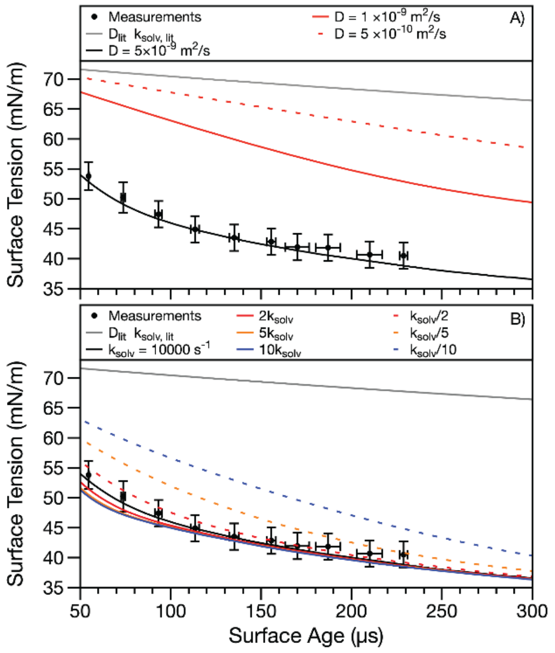

Figure S6: Sensitivity of model to input diffusion coefficient (D) and rate constants k_{solv} and k_{desolv} for 10 mM OTG. Black lines simulated with the parameters in Table 1. A) k_{solv} is held constant at 10,000 s^{-1} and D is reduced towards the expected diffusion coefficient of 5×10^{-10} m^2/s. B) D is held constant at 5×10^{-9} m^2/s and k_{solv} is varied by a factor of 2, 5 and 10. (k_{desolv} is also varied to keep the ratio K_{eq}^{surf} constant.) The grey line near the top of each panel shows the dynamics calculated with literature values for a generic surfactant, $D_{lit} = 5\times10^{-10}$ m^2/s and $k_{solv,\ lit} = 100$ s^{-1}.

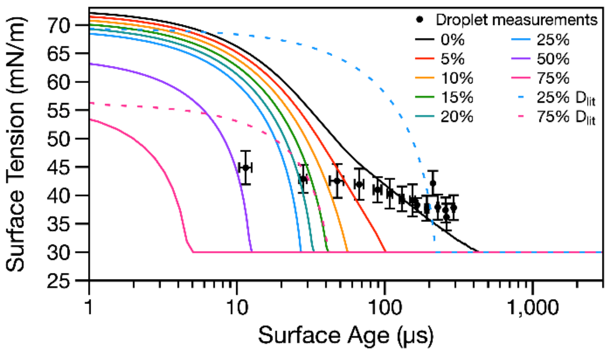

Figure S7: Simulated dynamic surface tension with 0 – 75% of the surface already covered in surfactant at time = 0 for 12 mM OTG (solid lines). Both the adsorption compartment and bulk concentration are assumed to be equal to the bulk concentration at t=0. In our experiments, the fraction of the droplet interface already formed as a meniscus at the droplet dispenser is about 25%. For 25 and 75% initial surface coverage at time = 0, the diffusion coefficient is also reduced to D_{lit} = $5\times10^{-10}m^2/s$ (dashed lines). Agreement is still not observed between the experimental data and the model, indicating that surfactant partitioning at the meniscus before droplet generation does not explain the discrepancy between the experimental data and modelling.

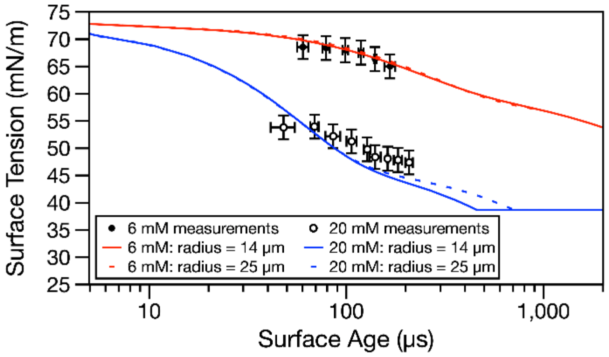

Figure S8: Kinetic model predictions for 6 mM (red) and 20 mM (blue) SDS in 14 µm (solid lines) and 25 µm (dashed lines) radius droplets. Droplet measurement datapoints represent an average of multiple experiments with droplet radii in the 14 – 25 µm range binned in time. Error bars in the x-direction represent the standard deviation of surface age in a bin. Error bars in the y-direction are the larger of the standard deviation of surface tension in a bin or 2.2 mN/m (the calculated measurement uncertainty from uncertainty in the droplet radius). The kinetic model predicts a maximum of 3 mN/m differences across the measured droplet size range for a concentration of 20 mM and are nearly indistinguishable at a concentration of 6 mM .

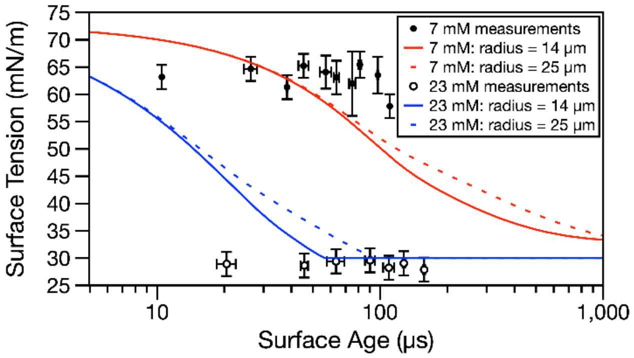

Figure S9: Kinetic model predictions for 7 mM (red) and 23 mM (blue) OTG in 14 µm (solid lines) and 25 µm (dashed lines). Droplet measurement datapoints represent an average of multiple experiments with droplet radii in the 14 – 25 µm range binned in time. Error bars in the x-direction represent the standard deviation of surface age in a bin. Error bars in the y-direction are the larger of the standard deviation of surface tension in a bin or 2.2 mN/m (the calculated measurement uncertainty from uncertainty in the droplet radius). The kinetic model predicts a maximum of 5 mN/m differences across the measured droplet size range for each concentration.

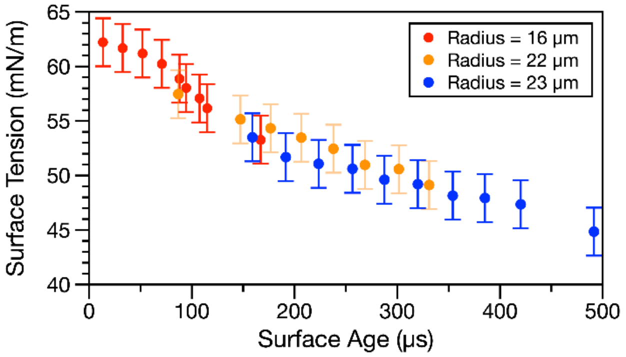

Figure S10: Dynamic surface tension of 10 mM SDS in 16 µm (red), 22 µm (orange) and 23 µm (blue) radius droplets. Overlap and continued trend across datasets for droplets of radius 16 µm, 22 µm and 23 µm agrees with the model predictions in Fig. S8 and S9.

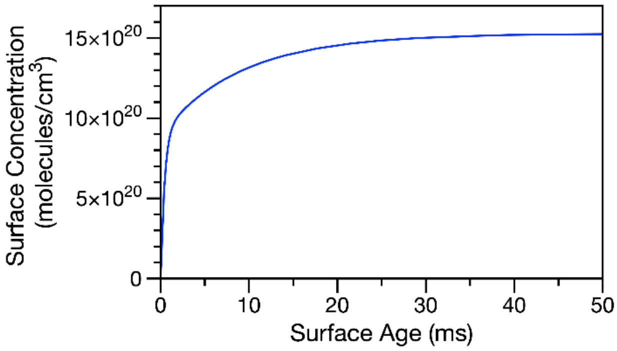

Figure S11: Predicted surface concentration of SDS adsorbed in the δ=1 nm thick surface of a 25 µm radius droplet containing 3 mM total SDS for the first 50 ms after surface formation.
